# Magnetic Polypyrrole-Gelatin-Barium Ferrite Cryogel as an Adsorbent for Chromium (VI) Removal

**DOI:** 10.3390/gels9100840

**Published:** 2023-10-23

**Authors:** Konstantin A. Milakin, Oumayma Taboubi, Jiřina Hromádková, Patrycja Bober

**Affiliations:** Institute of Macromolecular Chemistry, Czech Academy of Sciences, 162 00 Prague, Czech Republic; milakin@imc.cas.cz (K.A.M.); taboubi@imc.cas.cz (O.T.); hromadkova@imc.cas.cz (J.H.)

**Keywords:** adsorption, chromium, cryogel, polypyrrole, water purification

## Abstract

Polypyrrole-gelatin aerogels, containing magnetic barium ferrite (BaFe) particles, (PPy-G-BaFe) were synthesized by oxidative cryopolymerization and used as adsorbents for the removal of Cr(VI) from aqueous media. The removal was performed at pH 4, which was shown to be the optimal value, due to HCrO_4_^−^ being the dominant species in these conditions and its more favorable adsorption and reduction compared to CrO_4_^2−^, present at pH > 4. It was found that the presence of magnetic BaFe particles had no effect on the adsorption performance of PPy aerogels in terms of capacity and kinetics, which was attributed to its relatively low content in the composite. After the adsorption, the presence of chromium in the composites was confirmed by EDX and its electrostatic interaction with the adsorbent was pointed at by vibrational spectroscopy, corresponding to the accepted adsorption mechanism. The adsorption kinetics followed the pseudo-second-order model pointing at chemisorption being the rate-limiting step. The adsorption isotherm data was best fitting with the Temkin model. The maximum adsorption capacity, calculated using the Langmuir model, was 255.8 mg g^−1^ (the maximum experimental value was 161.6 mg g^−1^). Additionally, the possibility of Cr(VI) adsorption in the presence of Cl^−^, Br^−^, NO_3_^−^ and SO_4_^2−^ as interfering ions was shown.

## 1. Introduction

Chromium, in particular Cr(VI), is considered to be one of the most dangerous contaminants of environmental water due to its toxicity and carcinogenicity [1]. It can originate from natural sources (chromium-containing ore) [2] or be released into wastewater by leather [3], textile [4], mining and smelting [5] or electroplating [6] industries. According to the World Health Organization [7], the guideline value of chromium concentration in drinking water is estimated at 0.05 mg L^−1^, which is orders of magnitude lower than can be detected in environmental waters next to chromium-processing facilities [4]. Therefore, the development of new and the modification of existing materials and methods for purification of water from chromium-based contaminants is required.

Among the many methods for Cr(VI) removal from water, including membrane filtration [8], coagulation [9], solvent extraction [10], chemical precipitation [11], catalytic and photocatalytic reduction [12,13,14], etc., adsorption is considered one of the most attractive due to its simplicity, abundance of potential adsorbent precursors and possibility of adsorbent recyclability [15]. There are multiple classes of materials that have been shown to be effective adsorbents of Cr(VI), such as carbon derivatives from various sources [16,17,18], clays [19,20,21], zeolites [22,23,24] and conducting polymers, such as polyaniline and polypyrrole (PPy) [25,26,27,28,29].

Among the mentioned materials, PPy is a promising candidate for the Cr(VI) adsorption application due to its simple synthesis, environmental stability, suitable chemical structure with positively charged aromatic backbone, allowing both electrostatic interactions and hydrogen bonding, ability to undergo reversible doping–dedoping by pH changing [30]. However, as a result of the conventional chemical synthesis PPy is usually obtained as a powder [31], which can be difficult to remove from the treated solution, when used as an adsorbent. One of the ways of overcoming this problem can be the preparation of PPy cryogels with good mechanical integrity and handling properties [32,33,34,35] that are easily separated from liquid media. These materials can be prepared by oxidative one-step cryopolymerization in the presence of a water-soluble polymer, including gelatin [32,34], nanofibrillated cellulose [33], poly(vinyl alcohol) [35], methylcellulose [35], hydroxypropylcellulose [35] or poly(*N*-vinylpyrrolidone) [35], which acts as a stabilizer for improving mechanical properties. The macroporous structure and developed surface of PPy-based cryogels facilitate their application for water purification, which has been reported for adsorption of dyes [36], antibiotics [37,38] or Cr(VI) [33,35].

To the best of our knowledge, there are few works [33,35] describing the adsorption of Cr(VI) by PPy-based cryogels. Minisy et al. [33] prepared the cryogels by oxidative polymerization of pyrrole in the presence of a suspension of nanofibrillated cellulose in a frozen medium. After the optimization of the material composition, regarding nanofibrillated cellulose content, the highest measured adsorption capacity towards Cr(VI) was found to be 184 mg g^−1^. Bober et al. [35] prepared PPy-based cryogels by a similar crypolymerization procedure using various polymers, such as gelatin, poly(vinyl alcohol), hydroxypropylcellulose, methylcellulose and poly(*N*-vinylpyrrolidone) as stabilizers. The materials were used for the detailed study of their applicability as Cr(VI) adsorbents, including adsorption kinetics and isotherms. The calculated maximum adsorption capacities were reported to be in the range 221–498 mg g^−1^.

The novelty of the present manuscript lies in its attempt to make a further step for the improvement of applicability of PPy-based cryogels as adsorbents for removal of Cr(VI) from aqueous medium and facilitation of their separation after the adsorption, compared to the mentioned reports [33,35]. For this purpose, magnetic PPy-gelatin-barium ferrite (PPy-G-BaFe) cryogels were used in this work. The preparation of the cryogels by one-step oxidative cryopolymerization procedure, their characterization and application for the adsorption of dyes, such as Reactive Black 5, have been reported before [36]. They are the promising candidates for the water purification task due to the presence of magnetic BaFe particles, which enable their separation from the treated solution by magnetic field. Moreover, both BaFe [39] and gelatin [40,41] can also contribute towards the adsorption of Cr(VI) together with PPy. Thus, the prepared PPy-G-BaFe cryogels/aerogels were used for a systematic study of their applicability for adsorption of Cr(VI) from aqueous medium. The work included optimization of the adsorption conditions, study of kinetics models and adsorption isotherm, and for the first time assessment of the use of the PPy-based aerogels for adsorption in the presence of various interfering ions.

## 2. Results and Discussion

PPy-G-BaFe and PPy-G aerogels were prepared by oxidative cryopolymerization of pyrrole in the presence or in the absence of BaFe magnetic particles, respectively. The detailed characterization of the aerogels, including their morphology, composition, mechanical characteristics, conductivity, etc., has been previously reported [36]. The present work was focused on the applicability of the aerogels as adsorbents for removal of Cr(VI) from the aqueous medium.

### 2.1. Effect of pH on Cr(VI) Adsorption

It is known that adsorption of Cr(VI) on PPy-based materials is facilitated in acidic pH due to the distribution of various Cr(VI) forms across the pH range and the protonated state of the PPy backbone [42]. Moreover, at an acidic pH, the positive charges of gelatin chains, used as a stabilizer in PPy-G aerogels [43], and BaFe particles [39] can also contribute to the effect. Therefore, for the optimization of the Cr(VI) removal experiments, the adsorption of Cr(VI) from aqueous solution by PPy-G aerogels was performed at various pH levels with the focus on acidic conditions.

**Figure 1:**
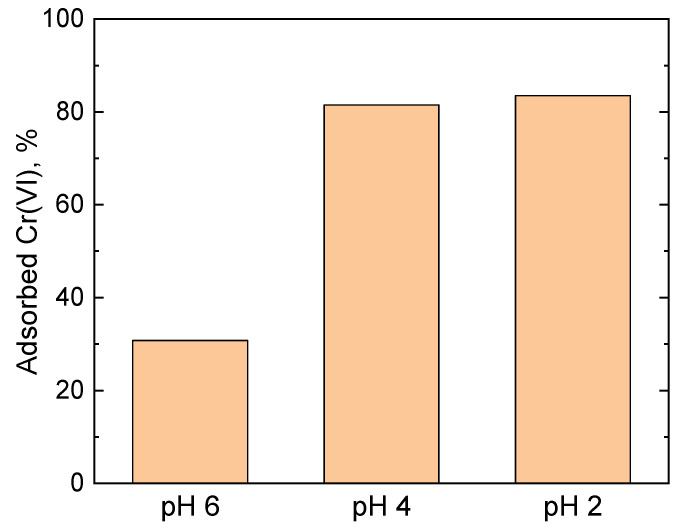
Fraction of the adsorbed Cr(VI) (35 mg L^−1^, 20 mL) by PPy-G aerogel (5 mg) at various pH levels after 24 h contact time (the corresponding UV–vis spectra are shown in Figure S1).

Figure 1 shows that with the decrease in the pH from 6 to 4 the fraction of the adsorbed Cr(VI) by the PPy-G aerogel increased from 31% to 82%, corresponding to an adsorption capacity of 43 and 114 mg g^−1^, respectively. A further decrease in the pH to 2 did not lead to a significant change of the adsorption performance of the materials, compared to pH 4, with the adsorbed Cr(VI) fraction and adsorption capacity of 84% and 117 mg g^−1^, respectively. The observed behavior can be attributed to the distribution of the Cr(VI) species in the solution at various pH levels. It is known that in the pH range from 2 to 4, HCrO_4_^−^ and Cr_2_O_7_^2−^ are the main species present in the solution. At a pH higher than 4, the concentrations of HCrO_4_^−^ and Cr_2_O_7_^2−^ in the solution start decreasing, while the concentration of CrO_4_^2−^ starts increasing, until around pH 8 where the fraction of HCrO_4_^−^/Cr_2_O_7_^2−^ reaches 0 and CrO_4_^2−^ becomes the main species in the solution [44]. The adsorption of HCrO_4_^−^ is more favorable compared to CrO_4_^2−^ due to HCrO_4_^−^ occupying fewer adsorption sites [42]. Moreover, as explained further in Section 2.7, the reduction of Cr(VI) by PPy chains is one of the processes involved in Cr(VI) removal. It is known [45] that HCrO_4_^−^ has higher redox potential than CrO_4_^2−^ (1.35 V and −0.13 V, respectively), hence it is a significantly stronger oxidant, which contributes to its removal from the solution by the reduction mechanism. These two factors, regarding the dominant presence of HCrO_4_^−^ in solution, presumably explain the observed increase in the adsorption capacity at pH 2 and 4, compared to pH 6. Based on the results, pH 4 was considered to be the optimal value for the adsorption studies and all further experiments were performed in these conditions.

### 2.2. Effect of the Presence of Magnetic BaFe Particles on Cr(VI) Adsorption

**Figure 2:**
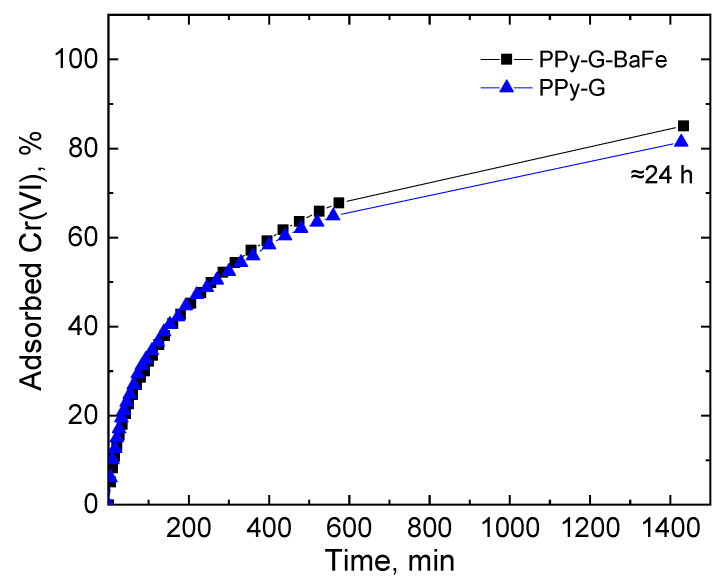
Kinetics of Cr(VI) adsorption (35 mg L^−1^, 20 mL, pH 4, 5 mg aerogel) by PPy-G and PPy-G-BaFe aerogels (the corresponding UV–vis spectra are shown in Figure S2).

The effect of incorporation of magnetic BaFe nanoparticles into the PPy-based aerogels on their performance towards the removal of Cr(VI) was assessed by the direct comparison of adsorption kinetics of PPy-G and PPy-G-BaFe. Figure 2 shows that PPy-G and PPy-G-BaFe aerogels had similar adsorption performance, which was not affected by the presence of BaFe magnetic particles. It is presumably attributed to its relatively low content (3.9 wt% [36]) in the aerogels). Thus, all further experiments were carried out using PPy-G-BaFe aerogels.

**Figure 3:**
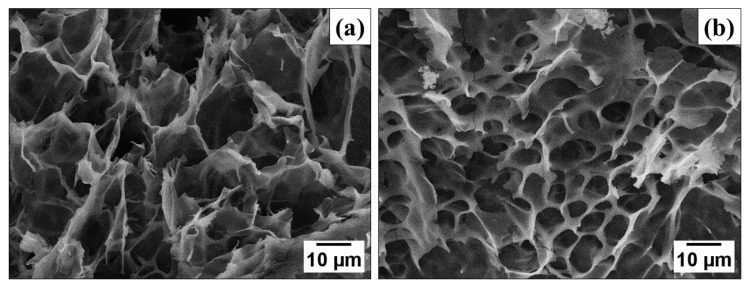
SEM image of PPy-G-BaFe aerogel (**a**) before and (**b**) after adsorption of Cr(VI).

PPy-G-BaFe aerogels after the adsorption of Cr(VI) were studied by SEM paired with EDX analysis to assess the incorporation of chromium into the materials and its effect on the aerogel morphology. Figure 3 shows that the macroporous morphology of PPy-G-BaFe aerogels was preserved after the adsorption, which can positively affect its potential reusability. The EDX analysis confirmed the presence of chromium (13.6 wt%) in the aerogel after the adsorption, which was absent in the initial material. However, it should be noted that EDX can only be used for semi-quantitative analysis of the chromium content in the material, due to the method being sensitive to local non-uniformities of the material and its limited penetration depth.

**Figure 4:**
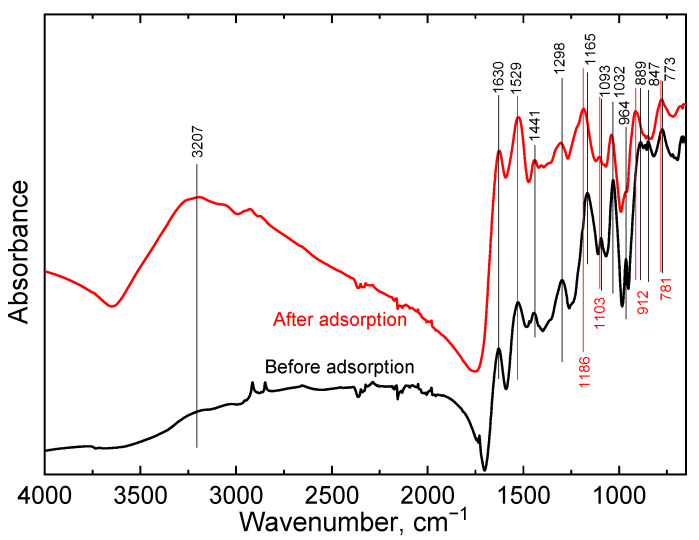
ATR-FTIR spectra of PPy-G-BaFe aerogels before and after Cr(VI) adsorption.

Vibrational spectroscopy was additionally used to study PPy-G-BaFe aerogels after adsorption of Cr(VI). In the ATR-FTIR spectrum (Figure 4) of PPy-G-BaFe, the characteristic peaks of PPy were observed as reported before [36], including 1529 cm^−1^ (pyrrole ring stretching), 1441 cm^−1^ (C–N stretching), 1298 cm^−1^ (C–H and C–N in-plane deformations), 1165 cm^−1^ (breathing vibrations of the pyrrole rings), 1093 cm^−1^ (N–H+ deformation), 964 cm^−1^ (out-of-plane C–C deformations of the pyrrole ring) and 773 cm^−1^ (C–H out-of-plane deformation). The carbonyl stretching band at 1630 cm^−1^ was attributed to gelatin.

After Cr(VI) adsorption, the bands 1165, 1093 and 773 cm^−1^, described above, shifted towards higher wavenumbers. The small peak at 889 cm^−1^ (C–H out-of-plane deformations), observed in the spectrum of pristine aerogels, shifted to 912 cm^−1^ and became more intense, which may have been due to the presence of HCrO_4_^−^ bound by electrostatic interaction [35]. Overall, the observed changes support the presence of interactions between the aerogels and Cr(VI) ions [46]. Moreover, the broadening of the N–H stretching vibration band, located at around 3207 cm^−1^ in the spectrum of the aerogel after adsorption, suggests that the amino group was involved in the adsorption process.

**Figure 5:**
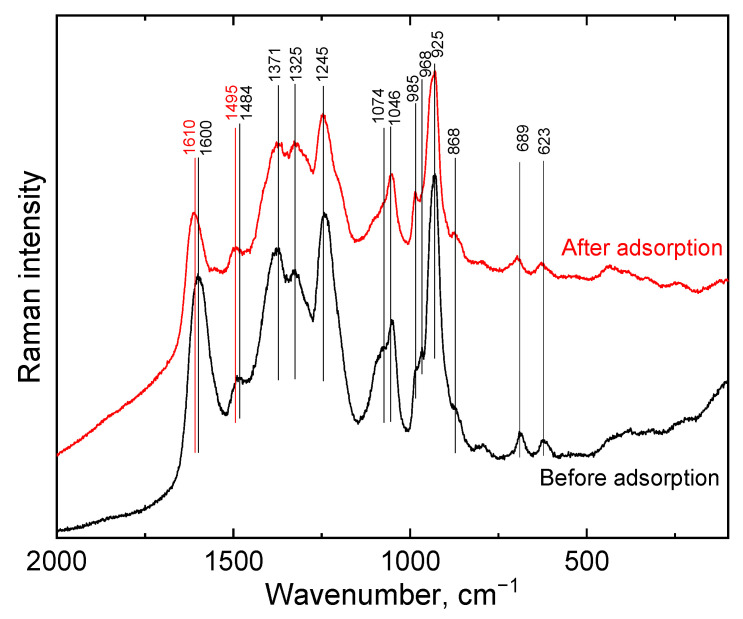
Raman spectra of PPy-G-BaFe aerogels before and after Cr(VI) adsorption, obtained at 830 nm excitation wavelength.

The Raman spectrum (Figure 5) of PPy-G-BaFe aerogel, obtained at 830 nm excitation wavelength, presents the characteristic bands of PPy as reported previously [36]. After Cr(VI) adsorption, the bands 1610 cm^−1^ and 1495 cm^−1^ were blue shifted. In addition, the change of the intensity of the two peaks situated at 1371 and 1325 cm^−1^, assigned to the ring stretching vibrations in the bipolaron and polaron/neutral fragments of PPy, corresponded to a less protonated state of PPy after the adsorption process [47].

### 2.3. Kinetics of Cr(VI) Adsorption

**Figure 6:**
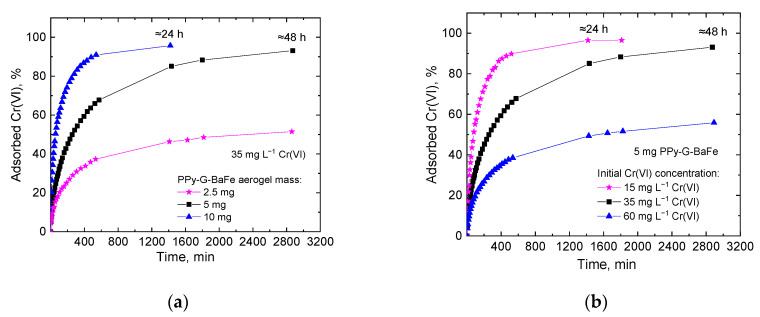
Kinetics of Cr(VI) adsorption by PPy-G-BaFe aerogel at (**a**) various masses of adsorbent (2.5, 5 and 10 mg aerogel, Cr(VI) 35 mg L^−1^, 20 mL, pH 4), (**b**) various initial Cr(VI) concentrations (Cr(VI) 15, 35 or 60 mg L^−1^, 20 mL, pH 4, 5 mg aerogel) (the corresponding UV–vis spectra are shown in Figure S3).

For the study of the kinetics of Cr(VI) removal by PPy-G-BaFe aerogels, the adsorption experiments were performed using various masses of the adsorbent and various initial Cr(VI) concentrations (Figure 6) and fitted using the pseudo-first-order and pseudo-second-order model equations.

The fitting parameters, provided in Table 1, show that the adsorption of Cr(VI) by PPy-G-BaFe aerogels followed the pseudo-second-order model. It is characterized by the higher *R*^2^ values, and its calculated *Q_e_* values were closer to the experimental parameters, compared to pseudo-first-order model. Therefore, it can be concluded that chemisorption is the rate-limiting step for the studied process [48].

### 2.4. Adsorption Isotherm

**Figure 7:**
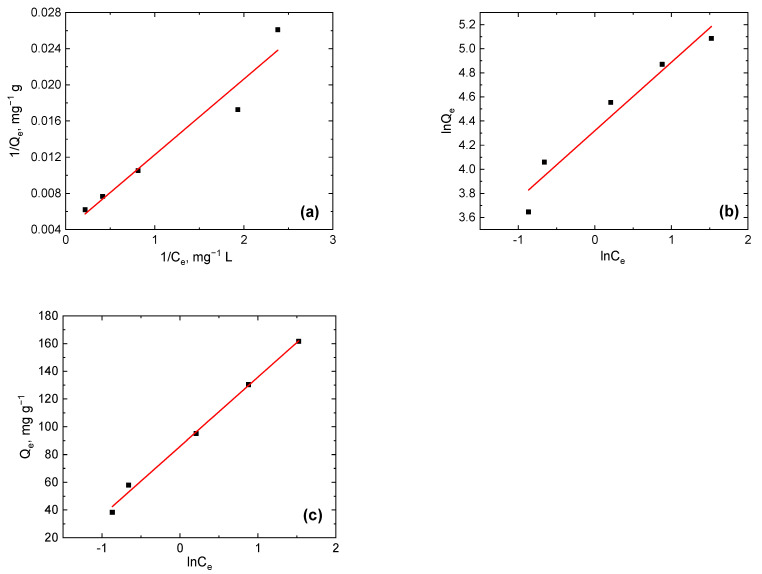
Fitting of adsorption isotherm, using linearized forms of (**a**) Langmuir, (**b**) Freundlich and (**c**) Temkin models, for adsorption of Cr(VI) (20 mL, pH 4) in the presence of PPy-G-BaFe aerogel (5 mg).

For the assessment of the mechanism of Cr(VI) adsorption by PPy-G-BaFe aerogel, adsorption isotherm, measured at various concentrations of Cr(VI) (the corresponding UV–vis spectra are shown in Figure S4), was fitted with Langmuir, Freundlich and Temkin isotherm models (Figure 7). The fitting parameters, provided in Table 2, show that the experimental data were best fitted by the Temkin model, which had the highest *R^2^* coefficient (0.996). This model describes the effect of indirect adsorption interactions, assuming that the heat of adsorption decreases linearly with the increase in the adsorbent surface coverage and uniform distribution of the binding energies [49]. The value of the coefficient 1/*n*, determined from the Freundlich model, close to 0.5 indicated the adsorption process being favorable (0.1 < *n* < 1.0) [50]. The maximum adsorption capacity determined from the Langmuir model was 255.8 mg g^−1^ (the maximum observed experimental value was 161.6 mg g^−1^), which is comparable to the PPy-based materials available in the literature (Table 3).

### 2.5. Adsorption of Cr(VI) in the Presence of Interfering Ions

The influence of various interfering ions on the Cr(VI) adsorption by PPy-G-BaFe aerogels was studied. Table 4 shows that the addition of 0.1 M of chloride, bromide or nitrate as interfering ions did not lead to a significant decrease in the adsorption capacity of the PPy-G-BaFe aerogels. The observed change of around 14–22% is likely attributed to the interference with electrostatic interactions of Cr(VI) with the adsorbent in these conditions. It can occur either due to the ionic strength-induced charge-shielding effect (decreased electrostatic attraction of the adsorbate and adsorbent due the change of the adsorbent surface potential) or competition of interfering anions with Cr(VI) for positively charged binding sites [59]. In the case of nitrate, its ability to form hydrogen bonds with the aerogels can also contribute to the effect. The influence of ionic strength on the aerogel adsorption performance was shown using various concentrations of Na_2_SO_4_. It was found that increasing the concentration from 0.033 to 0.1 M led to a decrease in the Cr(VI) adsorption capacity from 89 to 64 mg g^−1^, respectively, due to both a higher charge-shielding effect and adsorption site competition.

### 2.6. Regeneration of PPy-G-BaFe Aerogel after Adsorption of Cr(VI) and Its Reusability

**Figure 8:**
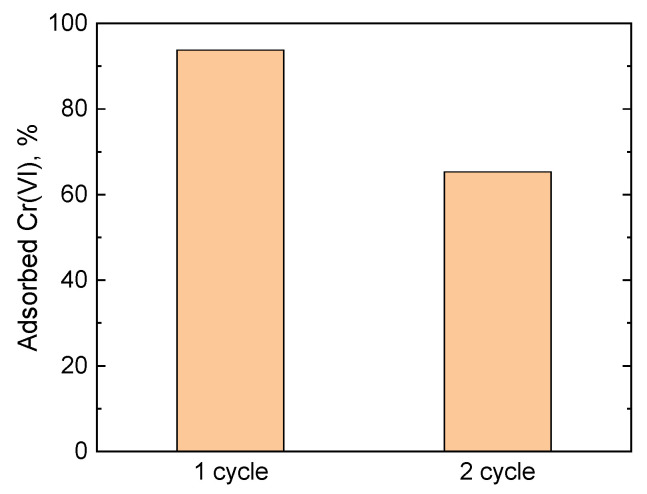
Fraction of adsorbed Cr(VI) (35 mg L^−1^, 20 mL, pH 4, 24 h, 5 mg aerogel) by PPy-G-BaFe aerogel at consecutive adsorption–desorption cycles (the corresponding UV–vis spectra are shown in Figure S6).

The possibility of PPy-G-BaFe aerogel reusability for Cr(VI) adsorption was studied through the adsorption–desorption cycle. The desorption step was based on deprotonation of PPy chains in alkaline conditions aimed to hinder electrostatic interactions of protonated PPy with negatively charged Cr(VI) ions [30]. After the desorption step, deprotonated PPy-G-BaFe aerogels were reprotonated in acidic conditions to restore positive charge on the conducting polymer chains and reactivate the Cr(VI) adsorption sites. Figure 8 shows that after the regeneration step the aerogel retained most of its adsorption performance, corresponding to 94% and 65% of removed Cr(VI) at the 1st and 2nd adsorption cycles, respectively. The observed difference might be attributed to incomplete chromium desorption after the 1st cycle, caused by partial PPy deprotonation at the desorption step in the studied conditions [60].

### 2.7. Mechanism of Cr(VI) Removal by PPy-G-BaFe

**Figure 9:**
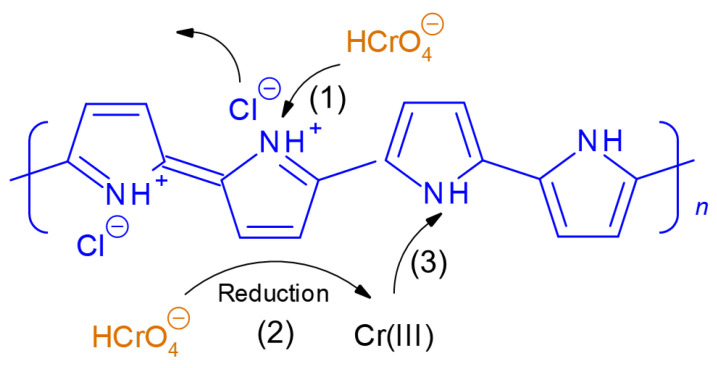
Interaction of Cr(VI) with PPy.

Based on the presented results and the literature data, the mechanism of Cr(VI) removal by PPy-G-BaFe aerogel involves the interaction of chromium species with all three components of the composite material. The interaction with PPy at pH 4 includes the following main processes [61,62] (Figure 9): (1) ion-exchange of HCrO_4_^−^ with PPy counterions (chloride anion) and its electrostatic binding to positively charged PPy chains, (2) reduction of HCrO_4_^−^ to Cr(III) by PPy and (3) binding of Cr(III) by neutral pyrrolic nitrogen atoms. Gelatin [40,43] and BaFe [39], which are both positively charged at pH 4, can also electrostatically bind HCrO_4_^−^, contributing to Cr(VI) removal.

## 3. Conclusions

Magnetic PPy-G-BaFe aerogels, prepared by one-step oxidative cryopolymerization, were shown to be efficient adsorbents of Cr(VI) from aqueous medium. The adsorption conditions were optimized in terms of pH, showing pH 4 as being the optimal value, based on the distribution of Cr(VI) species in the solution, and their relative adsorption favorability and oxidative properties. The efficiency of Cr(VI) removal was supported by SEM/EDX analysis, showing the presence of chromium in the macroporous structure of aerogels after the adsorption and vibrational spectroscopy, highlighting the electrostatic interaction between HCrO_4_^−^ anions and positively charged PPy chains. Despite the known ability of BaFe particles to adsorb Cr(VI), their presence in the aerogels did not affect the adsorption performance, presumably due to their relatively low content. The adsorption process followed the pseudo-second-order kinetics, corresponding to chemisorption as the rate-limiting step, and the Temkin isotherm model with maximum adsorption capacity 255.8 mg g^−1^ (Langmuir), which is comparable to the PPy-based materials available in the literature. The aerogels could be used for Cr(VI) adsorption in the presence of chloride, bromide, nitrate and sulfate as interfering ions without significant loss of adsorption capacity, depending on ionic strength, which can negatively affect the adsorption performance by both the charge-shielding effect and adsorption site competition.

## 4. Materials and Methods

### 4.1. Materials and Preparation

Pyrrole (98%, Sigma-Aldrich, Beijing, China), iron (III) chloride hexahydrate (≥99%, Sigma-Aldrich, Taufkirchen, Germany), gelatin (from porcine skin, Sigma-Aldrich, Taufkirchen, Germany), barium ferrite (BaFe, BaFe_12_O_19_, >97%, nanopowder, <100 nm particle size, St. Louis, MO, Sigma-Aldrich, USA), potassium dichromate (>99%, Sigma-Aldrich, St. Louis, MO, USA), sodium chloride (p.a., Lach-Ner, Neratovice, Czech Republic), sodium bromide (>98.5%, Lachema, Brno, Czech Republic), sodium sulfate (p.a., Lach-Ner, Neratovice, Czech Republic) and potassium nitrate (p.a., Lach-Ner, Neratovice, Czech Republic) were used as received.

PPy-G-BaFe cryogels were synthesized by oxidative crypolymerization of pyrrole by FeCl_3_ in the presence of BaFe dispersion in aqueous solution of gelatin, using the procedure reported before [36]. The added amount of BaFe nanoparticles was calculated to correspond to 10 wt%, relative to PPy in the resulting material. For the synthesis, 14 mmol of pyrrole and 0.122 g of BaFe particles were sonicated for 30 min in 35 mL of aqueous solution of gelatin (6 wt%) and left under mechanical stirring. The oxidant solution was prepared by dissolving 35 mmol of FeCl_3_·6H_2_O in 35 mL of gelatin solution (6 wt%). The monomer and oxidant solutions were mixed under vigorous stirring, transferred into plastic syringes, frozen in solid carbon dioxide/ethanol mixture and polymerized in a freezer at −24 °C for 7 days. After the polymerization and thawing of the cryogels at room temperature, they were removed from the molds, washed with water and freeze-dried to obtain aerogels.

PPy-G aerogels were prepared by the same procedure without BaFe particles.

### 4.2. Adsorption Study

K_2_Cr_2_O_7_ was used as a Cr(VI) source in the adsorption study. The concentration values presented in the work were Cr(VI) concentrations recalculated from the concentration of K_2_Cr_2_O_7_. The Cr(VI) adsorption was followed by the change of absorbance of a peak at 350 nm (368 nm for pH 6) using a Thermo Scientific Evolution 220 UV–vis spectrometer (Waltham, MA, USA). The spectra were measured without additional dilution. The adsorption experiments were performed at room temperature in the dark under constant mechanical shaking at 150 rpm.

The Cr(VI) removal fraction was calculated using the following equation, where *A*_350,*t*_ and *A*_350,0_ are absorbance values of the peak at 350 nm at the defined time, *t*, and the initial absorbance, respectively:

$$\frac{A_{350,0}-A_{350,t}}{A_{350,0}}\times 100\%$$

Adsorption capacity at the equilibrium (*Q_e_*) or at the defined time, *t*, (*Q_t_*) were calculated based on the equation, where *C*_0_ (mg L^−1^) is the initial Cr(VI) concentration, *C_e(t)_* (mg L^−1^) is the Cr(VI) concentration at the equilibrium (*e*) or the defined time (*t*), *m* (g) is the mass of the adsorbent and *V* (L) is the volume of the Cr(VI) solution:

$$Q_{e(t)}=\frac{{(C}_{0}-C_{e(t)})}{m}\times V$$

For the optimization of pH of the aqueous medium for the Cr(VI) adsorption experiments, 5 mg of PPy-G aerogels were brought into contact with Cr(VI) solution (35 mg L^−1^, 20 mL) with pH 2, 4 or 6 for 24 h.

The comparison between the adsorption performance of PPy-G and PPy-G-BaFe aerogels (5 mg) was performed in pH 4 using 20 mL, 35 mg L^−1^ Cr(VI) solution as the function of time for 24 h. After the experiment, PPy-G-BaFe aerogels were rinsed with water and freeze-dried for further investigation by vibrational spectroscopy and scanning electron microscopy.

The kinetics of Cr(VI) adsorption by PPy-G-BaFe aerogel was studied using various masses of the adsorbent (2.5, 5 and 10 mg of the aerogel) at the constant initial Cr(VI) concentration (35 mg L^−1^, 20 mL, pH 4) and various initial Cr(VI) concentrations (15, 35 and 60 mg L^−1^, 20 mL, pH 4) at the constant mass of the aerogel (5 mg). The chosen equilibrium times are shown in Table 5.

The linearized forms of pseudo-first-order and pseudo-second-order model equations were used for fitting of the kinetics experimental data:

$$\mathrm{Pseudo-first-order}\;\mathrm{equation}:\;\log\left(Q_{e}-Q_{t}\right)=\log Q_{e}-\frac{k_{1}}{2.303}t$$

$$\mathrm{Pseudo-second-order}\;\mathrm{equation}:\;\frac{t}{Q_{t}}=\frac{1}{k_{2}Q_{e}^{2}}+\frac{t}{Q_{e}}$$

In the shown equations, *Q_e_* and *Q_t_* (mg g^−1^) are the amounts of Cr(VI) adsorbed at the equilibrium and the defined time, *t* (min), respectively, *k*_1_ (min^−1^) is the pseudo-first-order rate constant, and *k*_2_ (g mg^−1^ min^−1^) is the pseudo-second-order rate constant [63].

Adsorption isotherm was measured at various initial concentrations of Cr(VI) solution (10, 15, 25, 35 and 45 mg L^−1^, 20 mL, pH 4) in the presence of 5 mg of PPy-G-BaFe aerogel. The equilibrium times for the experiments performed at different initial Cr(VI) concentrations were chosen as follows: 24 h for 10 and 15 mg L^−1^ of Cr(VI), 44 h for 25 mg L^−1^ of Cr(VI) and 48 h for 35 and 45 mg L^−1^ of Cr(VI). The linearized forms of Langmuir, Freundlich and Temkin model equations were used for fitting the experimental data:

$$\mathrm{Langmuir}\;\mathrm{model}:\;\frac{1}{Q_{e}}=\frac{1}{K_{L}Q_{max}^{}C_{e}}+\frac{1}{Q_{max}}$$

$$\mathrm{Freundlich}\;\mathrm{model}:\;\ln Q_{e}=\ln K_{F}+\frac{1}{n}\ln C_{e}$$

$$\mathrm{Temkin}\;\mathrm{model}:\;Q_{e}=B\ln K_{T}+B\ln C_{e}$$

In the provided equations, *K_L_* (L mg^−1^) is the Langmuir constant, associated with adsorption free energy, *Q_max_* (mg g^−1^) is the maximum adsorption capacity, *K_F_* is the Freundlich constant, related to adsorption capacity, *n* is the heterogeneity factor, *K_T_* is the Temkin isotherm constant, corresponding to the maximum binding energy, and *B* is the constant, related to the heat of adsorption [42,64].

For the study of adsorption of Cr(VI) by PPy-G-BaFe aerogels in the presence of interfering ions, 5 mg of the PPy-G-BaFe cryogel was placed into Cr(VI) solution (35 mg L^−1^, 20 mL, pH 4), containing NaCl (0.1 M), NaBr (0.1 M), Na_2_SO_4_ (0.033 M, 0.05 M or 0.1 M) or KNO_3_ (0.1 M), for 24 h.

For the study of PPy-G-BaFe aerogel reusability for Cr(VI) adsorption, the aerogel after Cr(VI) adsorption (5 mg of aerogel, 35 mg L^−1^ of Cr(VI), 20 mL, pH 4, 24 h) was placed into 0.003 M NaOH solution (50 mL). The NaOH solution was changed at least 3 times every 24 h. After the desorption step, the aerogel was washed with water and immersed into an HCl solution (0.01 M, 50 mL, 24 h). The reprotonated aerogel was rinsed with water and placed into the Cr(VI) solution (35 mg L^−1^, 20 mL, pH 4, 24 h) for the 2nd adsorption step.

### 4.3. Material Characterization Methods

The morphology of the freeze-dried materials was studied using a MAIA 3 Tescan high-resolution field emission gun scanning electron microscope (SEM, Brno, Czech Republic) at 3 kV. Before the measurement, the aerogel samples were cut at room temperature, fixed on the aluminum holders with a conductive double-sided carbon tape and covered with a thin carbon layer, using a JEE-4C JEOL vacuum evaporator (Tokyo, Japan). An energy-dispersive X-ray detector (EDX, X-MaxN 20, Oxford Instruments, Abington, UK) at 30 kV was used for assessment of the elemental composition of the investigated materials by standardless SEM/EDX analysis.

The FTIR spectra of the PPy-G-BaFe aerogels before and after Cr(VI) adsorption were obtained using attenuated total reflection (ATR) infrared spectroscopy with a Thermo Nicolet NEXUS 870 spectrometer (Waltham, MA, USA) equipped with an MCT nitrogen-cooled detector. The aerogel samples were placed onto the ZnSe crystal, and the ATR foot with a sharp tip ensured good contact between the sample and the crystal. The spectra were recorded at the resolution of 4 cm^−1^, and 256 scans were accumulated for each spectrum in the wavenumber range from 650 to 4000 cm^−1^. Raman spectra were recorded using a Renishaw inVia Qontor Raman Microscope (Wotton-under-Edge, UK) at 830 nm laser excitation wavelengths. The scattered light was analyzed with a spectrograph using holographic gratings of 1200 and 2400 lines mm^−1^, respectively. An ultra-high sensitive CCD camera registered the dispersed light.

## Figures and Tables

**Table 1:** Fitting parameters for pseudo-first-order and pseudo-second-order models for adsorption of various initial concentrations of Cr(VI) (20 mL, pH 4) on various masses of PPy-G-BaFe aerogel.

Cr(VI) Concentration, mg L^−1^	Adsorbent Mass, mg	*Q_e_* (Exp), mg g^−1^	Pseudo-First-Order			Pseudo-Second-Order		
			*k_1_*, min^−1^	*Q_e_*, mg g^−1^	*R^2^*	*k_2_*, g mg^−1^ min^−1^	*Q_e_*, mg g^−1^	*R^2^*
35	2.5	144.2	0.0015	106.6	0.963	4.12 × 10^−5^	147.9	0.994
35	5	130.4	0.0016	101.8	0.966	3.70 × 10^−5^	136.2	0.993
35	10	67.0	0.0052	46.6	0.976	0.00026	69.3	0.999
15	5	57.9	0.0052	44.6	0.980	0.00023	60.9	0.999
60	5	134.1	0.0013	99.1	0.955	3.79 × 10^−5^	137.6	0.992

**Table 2:** Fitting parameters for adsorption isotherms, plotted using linearized forms of Langmuir, Freundlich and Temkin models, for adsorption of Cr(VI) (20 mL, pH 4) in the presence of PPy-G-BaFe aerogel (5 mg).

Langmuir			Freundlich			Temkin		
*K_L_*, L mg^−1^	*Q_max_*, mg g^−1^	*R^2^*	*K_f_*	1n	*R^2^*	*K_T_*	*B*	*R^2^*
0.467	255.8	0.950	75.2	0.568	0.948	5.57	50.0	0.996

**Table 3:** Comparison of adsorption capacity (*Q*) values of various PPy-based materials, reported in the literature regarding adsorption of Cr(VI) from aqueous media and determined experimentally or from the Langmuir model.

Material	S_BET_, m^2^ g^−1^	*Q*, mg g^−1^	Method	Reference
PPy-nanofibrillated cellulose aerogel	50	184 (pH 2)	Experimentally	[33]
PPy-gelatin aerogel	-	337 (pH 2)	Langmuir	[35]
PPy-poly(vinyl alcohol) aerogel	-	498 (pH 2)	Langmuir	[35]
PPy-hydroxypropylcellulose aerogel	-	238 (pH 2)	Langmuir	[35]
PPy-methylcellulose aerogel	-	289 (pH 2)	Langmuir	[35]
PPy-poly(*N*-vinylpyrrolidone) aerogel	-	221 (pH 2)	Langmuir	[35]
PPy-natural pyrite composite	35	262 (pH 2)	Langmuir	[42]
PPy-cellulose acetate aerogel	-	183 (pH 2)	Langmuir	[51]
PPy-chitosan aerogel	235	401 (pH 2)	Langmuir	[52]
PPy-reduced graphene oxide-ethylenediamine tetraacetic acid Na salt aerogel	54	336 (pH 2)	Langmuir	[53]
PPy-graphene oxide aerogel	-	416 (pH 2)	Langmuir	[54]
PPy-polyethyleneimine-graphene oxide aerogel	-	478 (pH 2)	Langmuir	[55]
PPy-graphene oxide-iron nanoparticles	55	171 (pH 4)	Langmuir	[56]
PPy-cellulose sulfate fibers	0.14	198 (pH 2)	Langmuir	[57]
PPy-arginine-Fe_3_O_4_ nanocomposite	22	323 (pH 2)	Langmuir	[58]
PPy-G-BaFe aerogel	19 [36]	256 (pH 4)	Langmuir	This work

**Table 4:** Fraction of adsorbed Cr(VI) (35 mg L^−1^, 20 mL, pH 4, 24 h) by PPy-G-BaFe aerogel (5 mg) in the presence of NaCl (0.1 M), NaBr (0.1 M), Na_2_SO_4_ (0.033 M, 0.05 M and 0.1 M), KNO_3_ (0.1 M) and without interference (the corresponding UV–vis spectra are shown in Figure S5).

Interfering Compound	Concentration, M	Cr(VI) Fraction Adsorbed, %	Adsorption Capacity, mg g^−1^
None	-	84	118
NaCl	0.1	73	102
NaBr	0.1	74	103
KNO_3_	0.1	67	93
Na_2_SO_4_	0.1	46	64
Na_2_SO_4_	0.05	57	80
Na_2_SO_4_	0.033	63	89

**Table 5:** Equilibrium times chosen for the adsorption kinetics experiments using various masses of PPy-G-BaFe aerogel and various initial Cr(VI) concentrations.

Cr(VI) Concentration, mg L^−1^	Adsorbent Mass, mg	Equilibrium Time, h
35	2.5	48
35	5	48
35	10	24
15	5	24
60	5	48

## Data Availability

Data are available upon request.

## Supplementary Materials

The following supporting information can be downloaded at: https://www.mdpi.com/article/10.3390/gels9100840/s1, Figure S1: Evolution of UV–vis spectra of Cr(VI) solution (35 mg L^−1^, 20 mL, 5 mg aerogel) over time in contact with PPy-G aerogel at (**a**) pH 6, (**b**) pH 4 and (**c**) pH 2; Figure S2: Evolution of UV–vis spectra of Cr(VI) solutions (35 mg L^−1^, 20 mL, pH 4, 5 mg aerogel) over time in the presence of (**a**) PPy-G and (**b**) PPy-G-BaFe aerogels; Figure S3: Evolution of UV–vis spectra of Cr(VI) solutions (20 mL, pH 4) over time in the presence of PPy-G-BaFe aerogel at various masses of adsorbent (Cr(VI) 35 mg L^−1^) (**a**) 2.5 mg, (**b**) 5 mg, (**c**) 10 mg and at various initial Cr(VI) concentrations (5 mg aerogel) (**d**) 15 mg L^−1^ and (**e**) 60 mg L^−1^; Figure S4: Initial and equilibrium UV–vis spectra of Cr(VI) solutions (20 mL, pH 4, 5 mg aerogel) in the presence of PPy-G-BaFe aerogel at various initial Cr(VI) concentrations (**a**) 10 mg L^−1^, (**b**) 15 mg L^−1^, (**c**) 25 mg L^−1^, (**d**) 35 mg L^−1^ and (**e**) 45 mg L^−1^; Figure S5: Evolution of UV-vis spectra of Cr(VI) solution (35 mg L^−1^, 20 mL, pH 4, 5 mg aerogel) over time in contact with PPy-G-BaFe aerogel (**a**) without interference and in the presence of (**b**) 0.1 M NaCl, (**c**) 0.1 M NaBr, (**d**) 0.1 M KNO_3_, (**e**) 0.033 M Na_2_SO_4_, (**f**) 0.05 M Na_2_SO_4_ and (**g**) 0.1 M Na_2_SO_4_; Figure S6: Evolution of UV–vis spectra of Cr(VI) solution (35 mg L^−1^, 20 mL, pH 4, 5 mg aerogel) over time in the presence of PPy-G-BaFe aerogel at consecutive adsorption–desorption cycles: (**a**) 1st adsorption cycle and (**b**) 2nd adsorption cycle.
