# Supplementary material for: Magnetic Polypyrrole-Gelatin-Barium Ferrite Cryogel as an Adsorbent for Chromium (VI) Removal
Source: Gels. 2023 Oct 23;9(10):840. doi: 10.3390/gels9100840 (PMC10606301; doi:10.3390/gels9100840)
Supplement: Supplementary file 1 [file gels-09-00840-s001.zip › gels-2663598-supplementary.pdf]

gels MDPI

Supplementary Materials

Magnetic polypyrrole-gelatin-barium ferrite cryogel as an adsorbent for chromium (VI) removal

Konstantin A. Milakin, Oumayma Taboubi, Jiřina Hromádková and Patrycja Bober *

Institute of Macromolecular Chemistry, Czech Academy of Sciences, 162 00 Prague, Czech Republic; milakin@imc.cas.cz (K.A.M.); taboubi@imc.cas.cz (O.T.); hromadkova@imc.cas.cz (J.H.)

* Correspondence: bober@imc.cas.cz (P.B.)

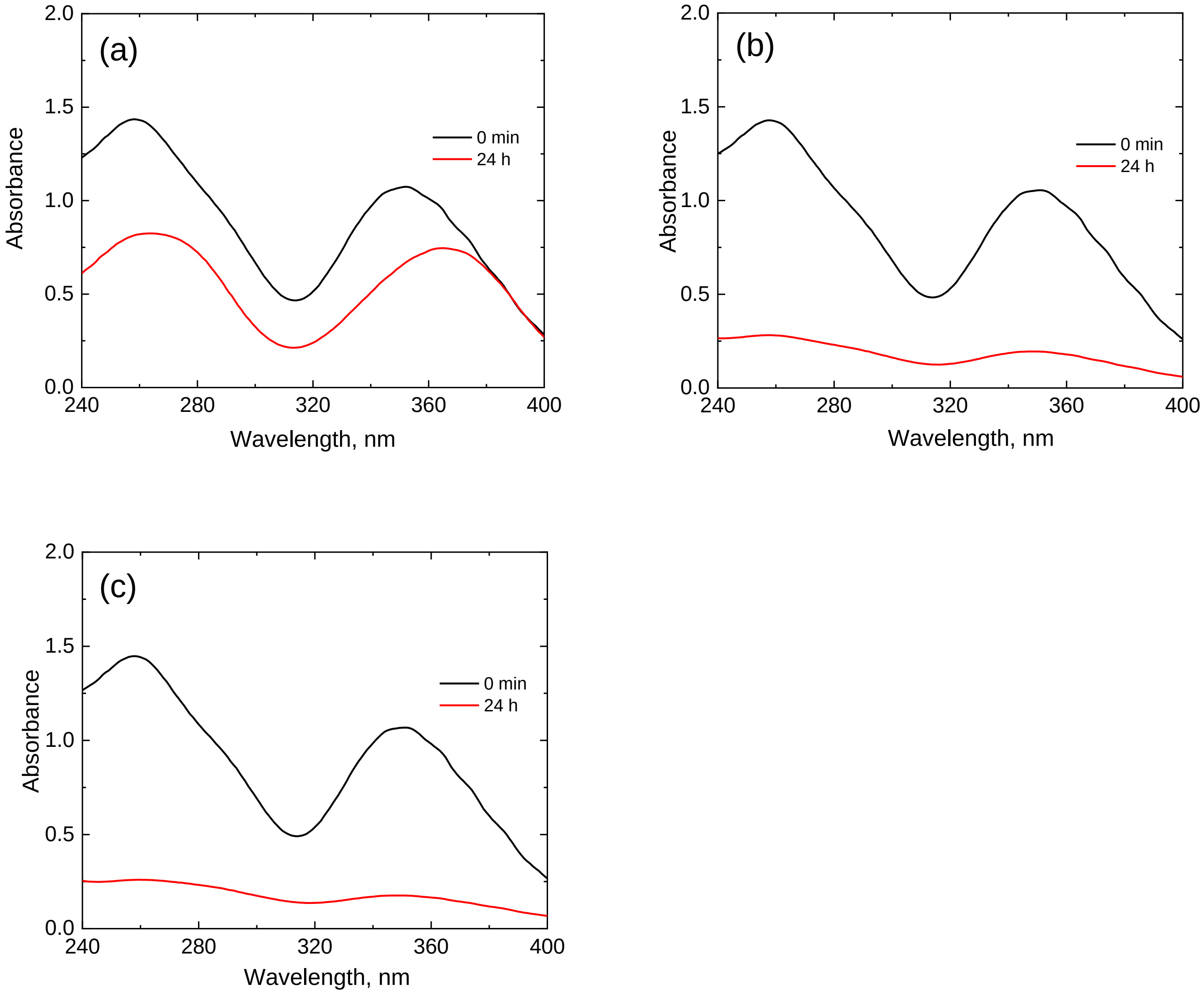

Figure S1. Evolution of UV-vis spectra of Cr(VI) solution (35 mg L^{-1}, 20 mL, 5 mg aerogel) over time in contact with PPy-G aerogel at (a) pH 6, (b) pH 4 and (c) pH 2.

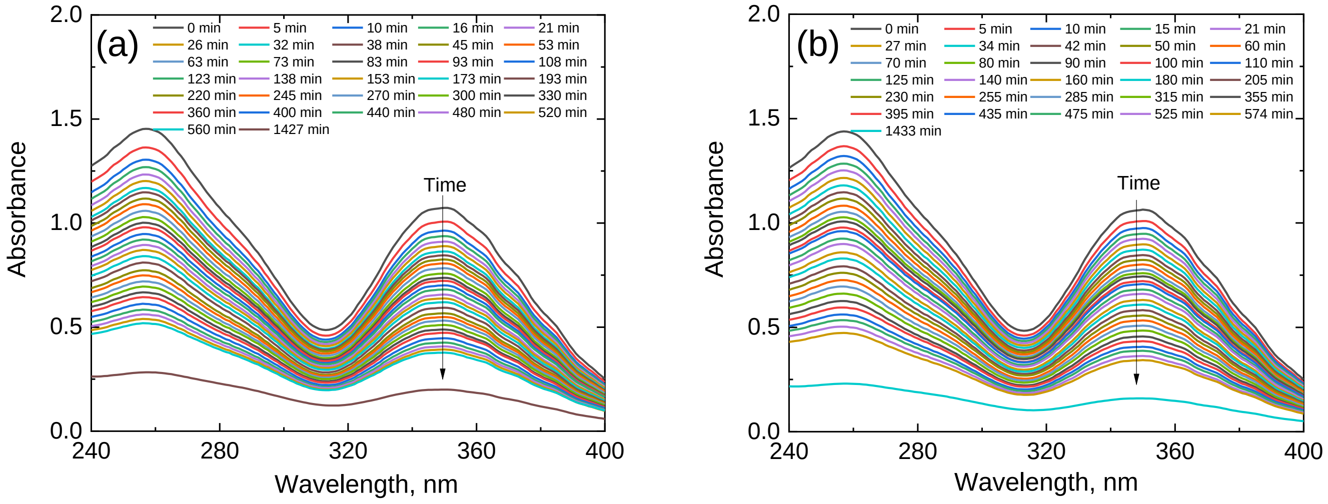

Figure S2. Evolution of UV-vis spectra of Cr(VI) solutions (35 mg L^{-1}, 20 mL, pH 4, 5 mg aerogel) over time in the presence of (a) PPy-G and (b) PPy-G-BaFe aerogels.

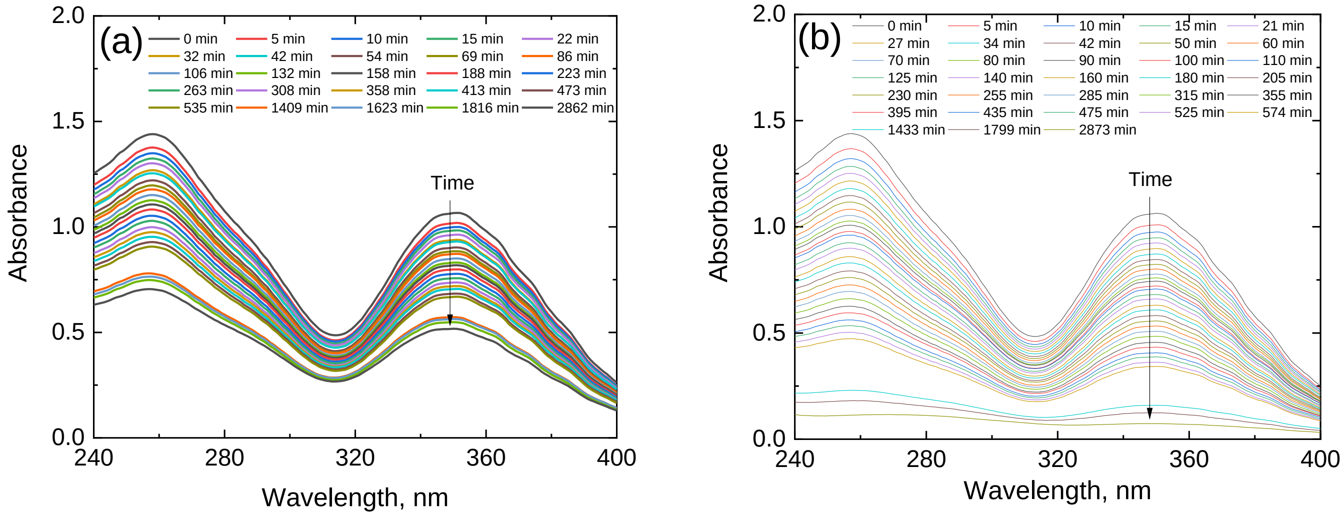

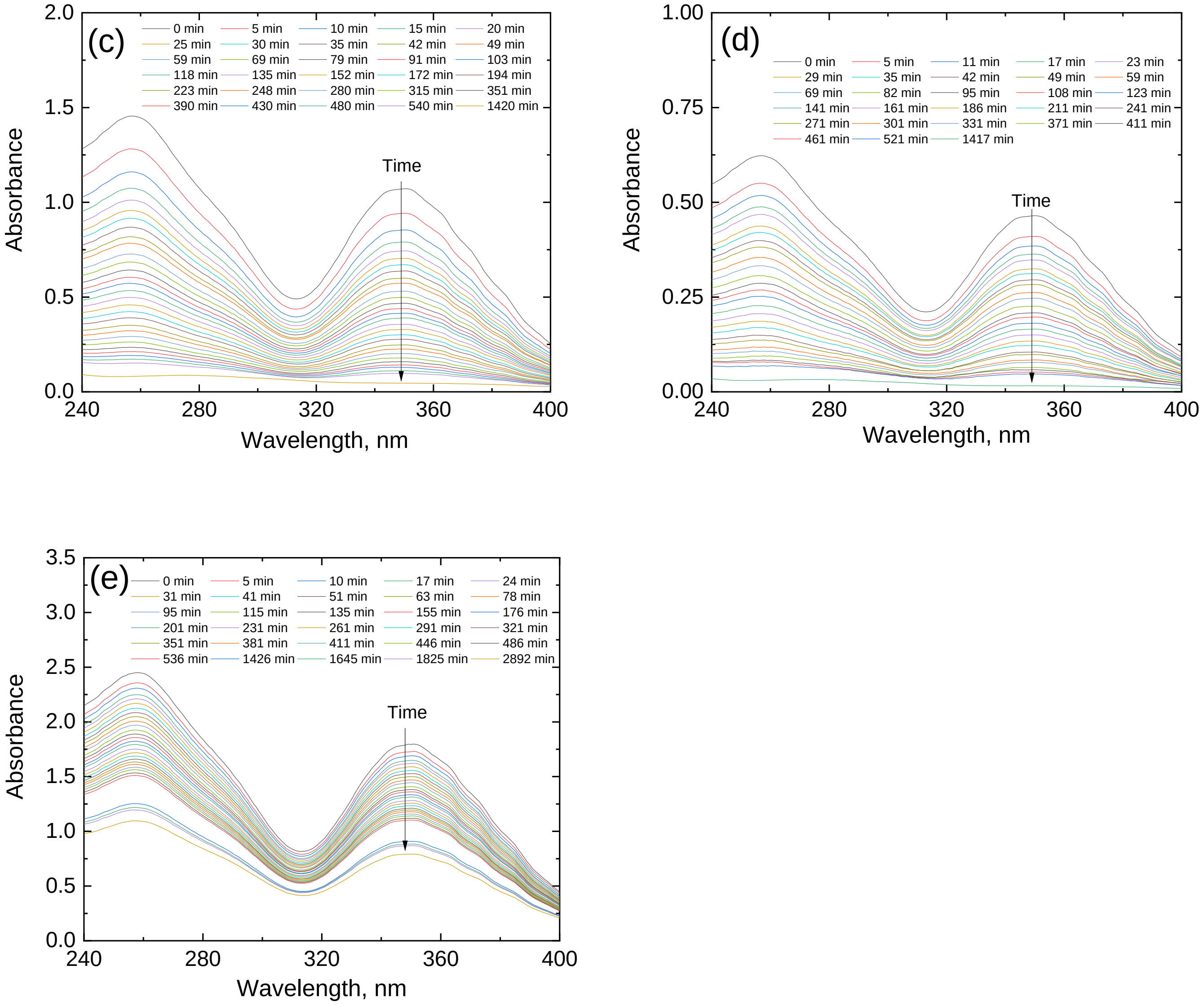

Figure S3. Evolution of UV-vis spectra of Cr(VI) solutions (20 mL, pH 4) over time in the presence of PPy-G-BaFe aerogel at various masses of adsorbent (Cr (VI) 35 mg L^{-1}) (a) 2.5 mg, (b) 5 mg, (c) 10 mg and at various initial Cr(VI) concentrations (5 mg aerogel) (d) 15 mg L^{-1} and (e) 60 mg L^{-1}.

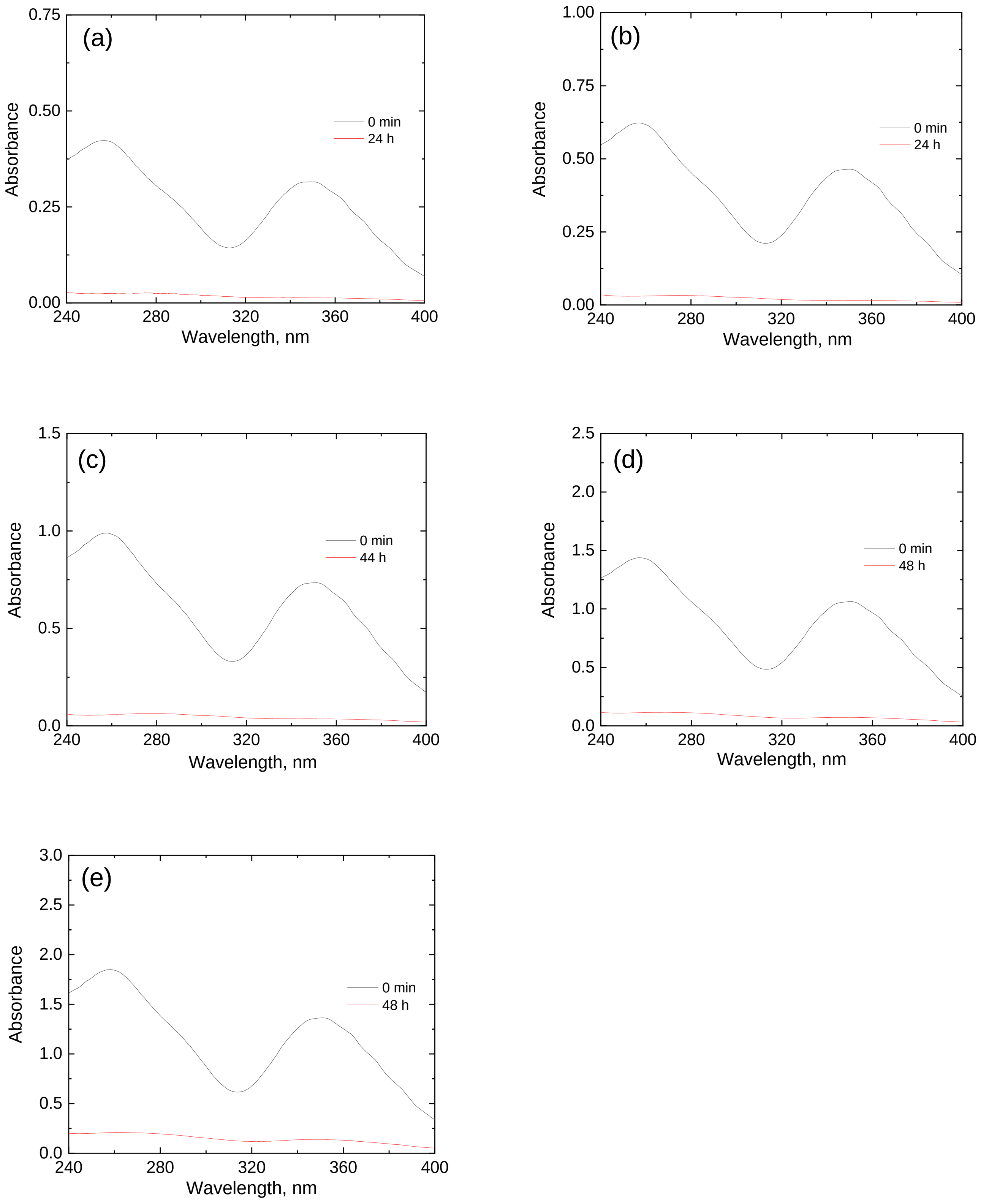

Figure S4. Initial and equilibrium UV-vis spectra of Cr(VI) solutions (20 mL, pH 4, 5 mg aerogel) in the presence of PPy-G-BaFe aerogel at various initial Cr(VI) concentrations (a) 10 mg L^{-1}, (b) 15 mg L^{-1}, (c) 25 mg L^{-1}, (d) 35 mg L^{-1} and (e) 45 mg L^{-1}.

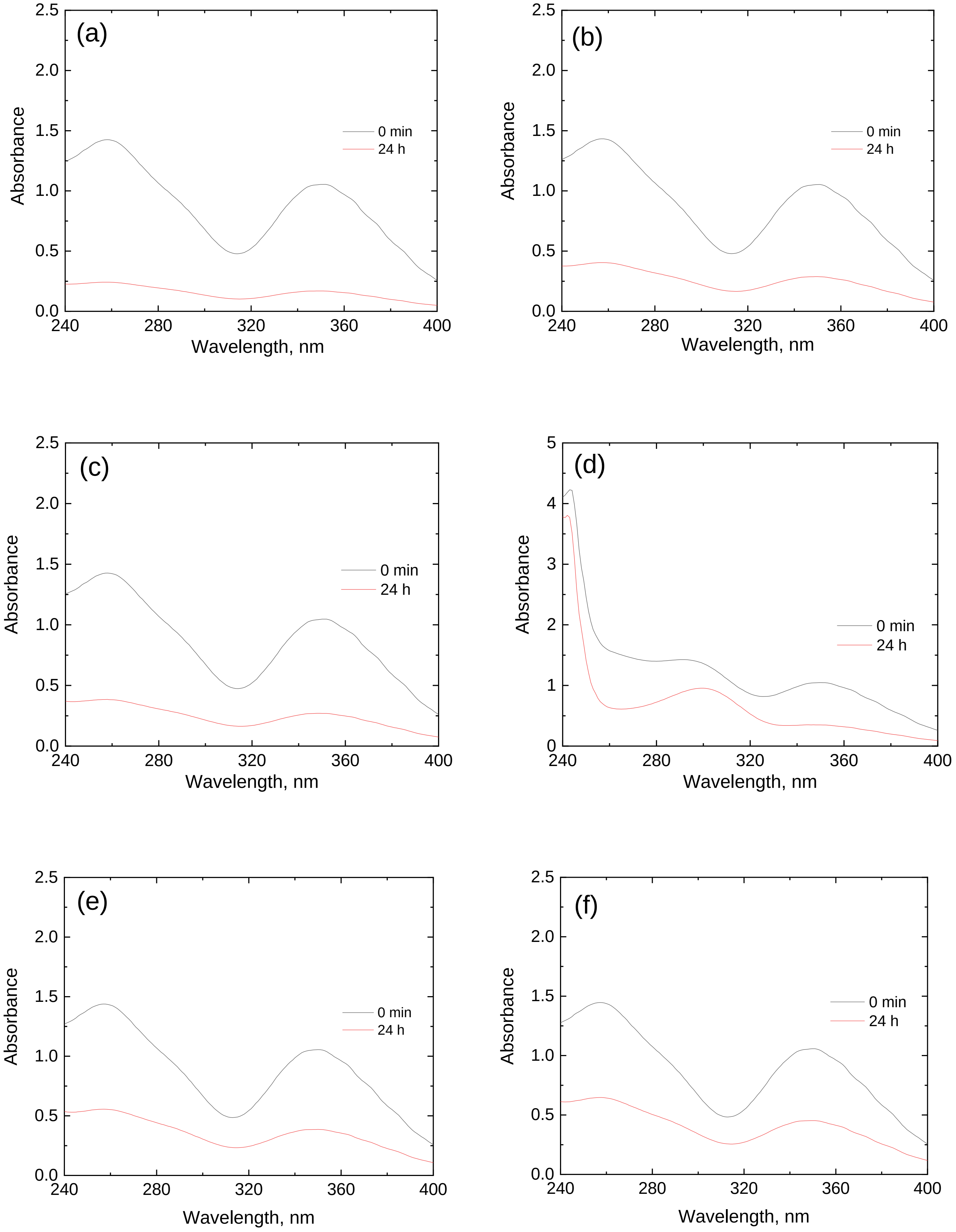
(a)
Absorbance
Wavelength, nm
0 min
24 h
2.5
2.0
1.5
1.0
0.5
0.0
240
280
320
360
400
(b)
Absorbance
Wavelength, nm
0 min
24 h
(c)
Absorbance
Wavelength, nm
0 min
24 h
(d)
Absorbance
Wavelength, nm
0 min
24 h
5
4
3
2
1
0
(e)
Absorbance
Wavelength, nm
0 min
24 h
(f)
Absorbance
Wavelength, nm
0 min
24 h

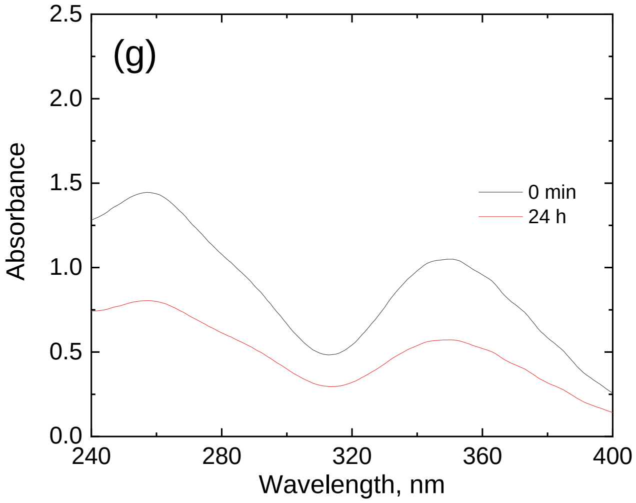

Figure S5. Evolution of UV-vis spectra of Cr(VI) solution (35 mg L^{-1}, 20 mL, pH 4, 5 mg aerogel) over time in contact with PPy-G-BaFe aerogel (a) without interference and in the presence of (b) 0.1 M NaCl, (c) 0.1 M NaBr, (d) 0.1 M KNO_3, (e) 0.033 M Na_2SO_4, (f) 0.05 M Na_2SO_4 and (g) 0.1 M Na_2SO_4.

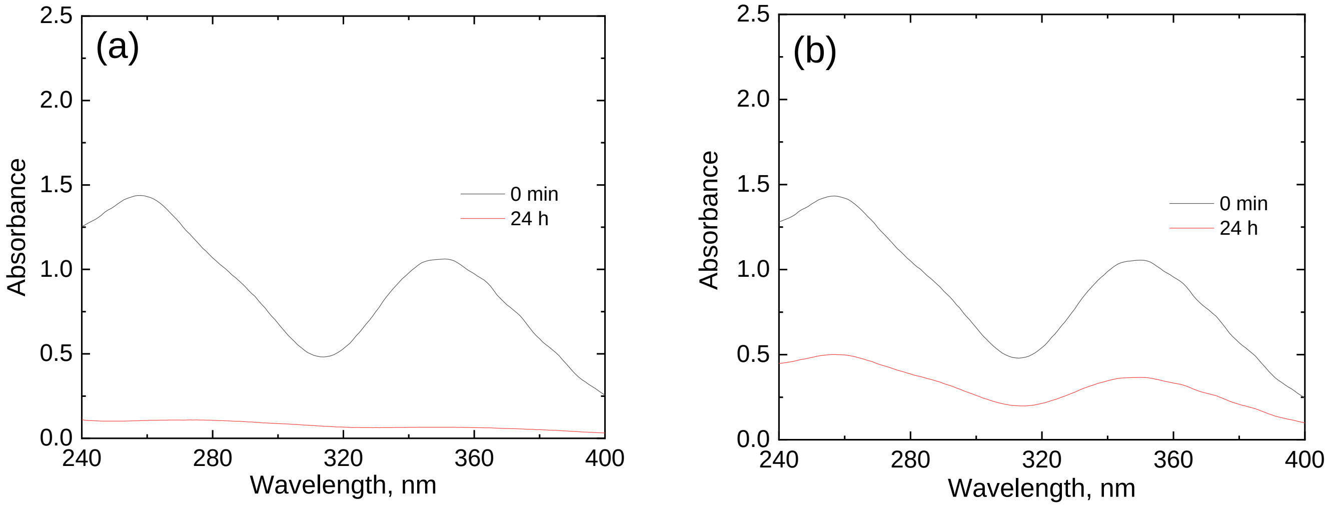

Figure S6. Evolution of UV-vis spectra of Cr(VI) solution (35 mg L^{-1}, 20 mL, pH 4, 5 mg aerogel) over time in the presence of PPy-G-BaFe aerogel at consecutive adsorption-desorption cycles: (a) 1st adsorption cycle and (b) 2nd adsorption cycle.
